# Accuracy of artificial intelligence software for CT angiography in stroke

**DOI:** 10.1002/acn3.51790

**Published:** 2023-05-19

**Authors:** Grant Mair, Philip White, Philip M. Bath, Keith Muir, Chloe Martin, David Dye, Francesca Chappell, Rüdiger von Kummer, Malcolm Macleod, Nikola Sprigg, Joanna M. Wardlaw, for the RITeS Collaboration

**Affiliations:** ^1^ Centre for Clinical Brain Sciences University of Edinburgh Edinburgh UK; ^2^ Translational and Clinical Research Institute, Newcastle University, Newcastle Upon Tyne Hospitals NHS Trust Newcastle upon Tyne UK; ^3^ Stroke Trials Unit, Mental Health & Clinical Neuroscience University of Nottingham Nottingham UK; ^4^ Institute of Neuroscience & Psychology, University of Glasgow Glasgow UK; ^5^ Department of Neuroradiology University Hospital, Technische Universität Dresden Dresden Germany; ^6^ UK Dementia Research Institute Centre at the University of Edinburgh Edinburgh UK

## Abstract

**Objective:**

Software developed using artificial intelligence may automatically identify arterial occlusion and provide collateral vessel scoring on CT angiography (CTA) performed acutely for ischemic stroke. We aimed to assess the diagnostic accuracy of e‐CTA by Brainomix™ Ltd by large‐scale independent testing using expert reading as the reference standard.

**Methods:**

We identified a large clinically representative sample of baseline CTA from 6 studies that recruited patients with acute stroke symptoms involving any arterial territory. We compared e‐CTA results with masked expert interpretation of the same scans for the presence and location of laterality‐matched arterial occlusion and/or abnormal collateral score combined into a single measure of arterial abnormality. We tested the diagnostic accuracy of e‐CTA for identifying any arterial abnormality (and in a sensitivity analysis compliant with the manufacturer's guidance that software only be used to assess the anterior circulation).

**Results:**

We include CTA from 668 patients (50% female; median: age 71 years, NIHSS 9, 2.3 h from stroke onset). Experts identified arterial occlusion in 365 patients (55%); most (343, 94%) involved the anterior circulation. Software successfully processed 545/668 (82%) CTAs. The sensitivity, specificity and diagnostic accuracy of e‐CTA for detecting arterial abnormality were each 72% (95% CI = 66–77%). Diagnostic accuracy was non‐significantly improved in a sensitivity analysis excluding occlusions from outside the anterior circulation (76%, 95% CI = 72–80%)*.*

**Interpretation:**

Compared to experts, the diagnostic accuracy of e‐CTA for identifying acute arterial abnormality was 72–76%. Users of e‐CTA should be competent in CTA interpretation to ensure all potential thrombectomy candidates are identified.

## Introduction

Among patients presenting acutely with ischemic stroke, CT angiography (CTA) is commonly used to identify candidates for thrombectomy. This CTA is principally used to identify proximal intracranial arterial occlusion in the internal carotid and middle cerebral arteries (ICA and MCA, respectively) and the vertebral and basilar arteries, collectively referred to as LVOs (large vessel occlusions). In addition, for patients with LVO, CTA can be used to determine the extent of collateral blood supply reaching affected brain.^1^ Thus, CTA provides some measure of ischemic brain tissue viability.^2^

Since in most patients the potential benefit of thrombectomy diminishes with time,^3^ acute stroke CTA should be acquired, interpreted and acted upon rapidly. However, expertise for immediate interpretation of stroke CTA is not available 24/7, nor is it necessarily widely distributed outside comprehensive stroke centres.^4^

Software developed using artificial intelligence (AI) designed to assist clinicians interpret stroke CTA and detect LVO are increasingly available. However, in this rapidly evolving field of radiology AI, standardised testing methods do not yet exist, and peer‐reviewed evidence of AI software efficacy is only beginning to emerge.^5^

e‐CTA is one software application developed using AI to automate the identification of distal ICA or proximal MCA occlusion and to score anterior circulation arterial collaterals on CTA. The software is cleared for clinical use (currently only for collateral quantification in the US), but there is limited peer‐reviewed testing of e‐CTA.^6^, ^7^, ^8^ Our study aims to independently assess the diagnostic accuracy of e‐CTA for detecting arterial abnormality in acute ischemic stroke.

## METHODS

### Study design

We used prospectively collected data from clinical trials and observational studies of acute stroke in which CTA had been assessed by panels of masked experts (reference standard). We compared e‐CTA (Brainomix™ Ltd) software results (index test) with the previously acquired expert interpretation of the same scans. Expert interpretation was completed before the index test was applied.

We conducted analyses in two ways:

1. Using all patients in a clinically representative cohort
2. To comply with the manufacturer guidance for use of e‐CTA software, we conducted a sensitivity analysis in a subset of patients excluding those with non‐ICA‐MCA occlusion and where CT slice thickness was >1 mm.

We report our results according to STARD (Standards for Reporting Diagnostic Accuracy Studies).^9^

### Patient population

We have previously described the RITeS (Real‐world Independent Testing of e‐ASPECTS Software) study,^10^, ^11^ a collaboration of up to 10 multicentre national and international acute stroke studies that recruited patients within 6 h of symptom onset. For the present analysis, we identified all patients in RITeS with CTA acquired at baseline. Six of nine RITeS studies that recruited patients with ischemic stroke collected (single phase) CTA for some participants. These six studies include five randomised‐controlled trials (two testing thrombolytics, one testing thrombectomy, one testing imaging strategies and one testing blood pressure lowering) and one prospective observational study (assessing glycaemic control).^12^, ^13^, ^14^, ^15^, ^16^, ^17^ All six studies centrally recorded patient demographics, stroke severity (National Institutes of Health Stroke Scale, NIHSS), time elapsed from stroke onset to imaging, treatment allocation and 3–6 months functional outcome collected by central masked researchers. All six studies individually obtained research ethics committee approval. Consent was acquired from or on behalf of all recruited patients.

### Sample for e‐CTA evaluation

We have previously described our method for deriving a ‘real‐world’ clinically representative dataset from all patients available to RITeS.^10^, ^11^ In this analysis of e‐CTA, we:

1. Aimed for age, sex, stroke severity and time since symptom onset similar to results provided by UK national audit (SSNAP, April 2018–March 2019, www.strokeaudit.org), and pooled randomised‐controlled trial data.^1^, ^18^ We expected RITeS patients to be within the ranges presented by the other datasets.^10^, ^11^ If necessary, we used stratified random sampling to modify the case mix.
2. Did not exclude imaging based solely on quality, if intracranial arterial occlusion involved vessels other than the ICA‐MCA axis, or if the centrally adjudicated final diagnosis (derived using all available clinical and imaging data) was not ischemic stroke (a small number of patients were given a final diagnosis of stroke mimic).

### Expert Image assessment

Prior to RITeS, baseline CTA in the original six studies was rated by central expert panels (total 15 readers with some overlap between trials: 11 neuroradiologists/neurointerventionists and 4 neurologists with extensive experience reviewing stroke CTA, range 5–25+ years, from 10 centres), masked to other clinical data except the concurrent non‐enhanced brain CT, and in two of the smaller studies, the side affected by stroke symptoms (186/668, 28%). Each scan was rated by a single expert. When maximum intensity projection (MIP) views were produced by recruiting sites, these were also provided to experts. CTA was scored for the presence, extent and location of arterial occlusion by named vessel and the extent of any collateral supply.^19^ All visible intracranial arteries were assessed (i.e. including anterior and posterior circulations, and including LVO and more distal non‐LVO vessels). For the current analysis, we considered ICA and the first MCA segment (M1) proximal, and more distal MCA segments (M2+) distal. Collateral extent was assessed using a 3‐point method described by Miteff.^20^ CTA image quality was recorded as good, moderate or poor based on patient position, artefacts and completeness of coverage. CTA assessment for four of six RITeS studies (comprising >70% of the patients included here) was conducted identically by 12 of our 15 experts using a clinically validated online platform (SIRS, https://sirs2.ccbs.ed.ac.uk/sirs2).^19^ We have previously tested inter‐rater agreement for seven experts using SIRS and CTA data from one of the same trials used in RITeS.^13^ Krippendorff's Alpha was 0.70 (substantial agreement) for the identification of angiographic occlusion (included LVO and non‐LVO) and 0.56 (moderate agreement) for collateral scoring.^21^

### Image software processing

We processed CTA in batches of 10 scans using the DICOM (Digital Imaging and Communications in Medicine) format on the cloud‐based Brainomix platform (https://brainomix.com, version 9). Per patient, we selected the earliest CTA after stroke and to best meet software specifications, uploaded the thinnest image slices available over a secure connection for automated processing. Brainomix was not involved in the image processing.

We recorded all upload and processing outcomes. We considered processing to be successful when software provided an LVO detection result and/or a collateral score. Where a CTA did not process on first attempt, we made at least one further attempt. e‐CTA allows users to input the side affected by stroke. We manually included this information for a subset (50%, 274 of 545 CTAs successfully processed) where side information was available. We exported e‐CTA results to spreadsheets for analysis. We did not review e‐CTA imaging overlays for every case but inspected batched outputs during processing. We also reviewed imaging overlays when uploading affected side data, and in cases that did not process at first attempt.

Once CTA processing was complete, we randomly selected a subsample of 20 CTAs stratified by study that had been successfully processed by e‐CTA, for repeatability testing. To ensure e‐CTA did not recognise scans by their DICOM headers at repeat testing (and merely present the original results), we created new unique scan identifiers for this subsample using modiCAS DICOM anonymiser (Erlangen, Germany). At the time of repeat testing, e‐CTA had been upgraded to version 10. Therefore, we also repeated analysis of the 20 original CTAs using version 10.

### Primary outcomes

Compared with masked experts:

1. Intracranial arterial occlusion detected by e‐CTA.
2. MCA collateral scores provided by e‐CTA.
3. Diagnostic accuracy of e‐CTA for detecting any intracranial arterial abnormality (combined LVO detection and collateral scoring)

### Secondary outcomes

1. Factors associated with e‐CTA processing success and diagnostic accuracy.
2. Repeatability of e‐CTA results on a subset of CTAs presented twice.

### Statistics

We summarise here the RITeS Statistical Analysis Plan.^10^

We followed an ‘intention‐to‐analyse’ basis for testing, equivalent to the ‘intention to treat’ principle, which included all cases regardless of whether scan processing was successful or not. We included the laterality of abnormalities in all comparisons.

For univariate testing, we used *χ* ^2^ to compare proportions and Mann–Whitney *U* to compare non‐parametric continuous or ordinal data. For multivariable testing, we pre‐specified variables^10^ and checked for multi‐collinearity (variance inflation factors >5).

For diagnostic accuracy testing of e‐CTA, we used the masked expert opinion of CTA as the reference standard and assigned cases as true/false positive/negative based on software identifying any angiographic abnormality (LVO detection and/or an abnormal collateral score combined) compared with experts. Cases were considered false positive (rather than false negative) where software and experts disagreed on the side affected, that is we have focussed on the software result. We also present diagnostic accuracy results for (1) LVO detection alone (for easier comparison with previous work) and, (2) when information on the side affected by stroke is provided versus not provided. We derived sensitivity, specificity, and accuracy (with Wilson score derived confidence intervals)^22^ using both raw data, and in a random‐effects meta‐analysis model of individual patient data (one‐step meta‐analysis) to provide (1) overall estimates of sensitivity and specificity, (2) to assess variation within/between the contributing studies in RITeS and (3) to account for individual study result clustering. To aid clinical understanding and real‐world applicability, we summarised diagnostic accuracy results with normalised frequencies as proportions per 100 patients imaged.^23^

e‐CTA uses a modified 4‐point Tan method to score collateral extent.^24^ To compare the 4‐point Tan CTA collateral score (e‐CTA) and the 3‐point Miteff score (used by study experts),^20^ we tested all possible combinations for reducing the 4‐point Tan to a 3‐point score, and we selected the combination with the best expert‐software agreement.

For repeatability testing, we compared initial vs repeat results for the presence/location of occlusion and collateral scores.

We did not impute but report missing data.

We used SPSS, IBM Corporation (Armonk, USA) for analyses, unless otherwise stated. We preferentially report 95% CI, but where appropriate, report *P*‐values.

## RESULTS

### Representative dataset

**Figure 1:**
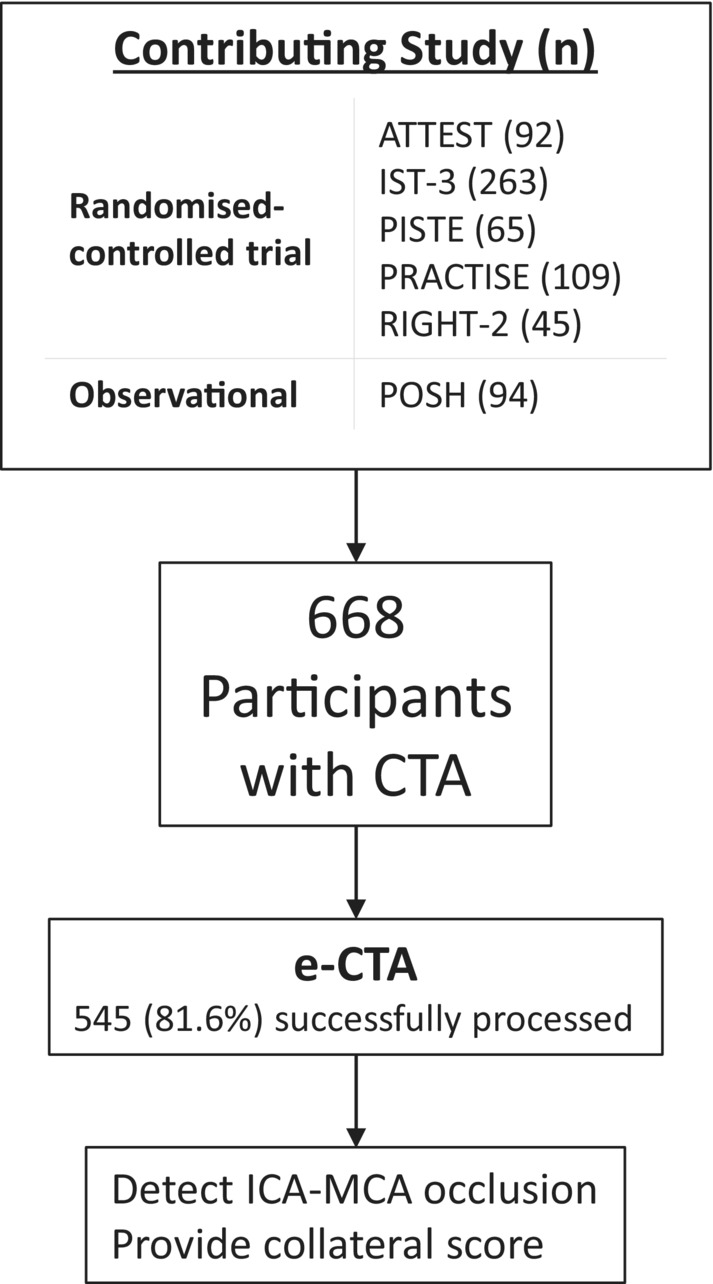
Flowchart of individual study patient contribution and CTA image processing in RITeS.

We include 668 patients with CTA (Fig. 1) 332 (50%) female, median age 71 years. Final diagnosis was either ischemic stroke (640, 96%) or stroke mimic (28, 4%). Median NIHSS 9. Median time elapsed since stroke onset 2.3 h. From potentially eligible patients, 412/640 (64%) were treated with intravenous thrombolytic and where available 34/66 (52%) were treated with thrombectomy (only 2 trials in RITeS had any thrombectomy). RITeS clinical variables were similar to the pre‐specified comparative datasets and thus considered clinically representative without the need for stratification (Table S1).

Table 1 details the radiological features of the 668 CTA scans according to experts. Of arterial occlusions identified, most affected the anterior circulation (343/365, 94%). From 341 ICA/MCA occlusions, 256 (75%) were proximal (ICA or M1), 85 (25%) were distal (M2+). Expert collateral scoring was available for 583/668 (87%) scans. Of available collateral scores, most were ‘good‐moderate’, 491/583 (84%). Of patients with a final diagnosis of stroke mimic, none had an alternative structural cause for symptoms. Most scans were good‐moderate quality (94%) with thin image slices (89%, ≤1 mm). CTAs were acquired between 2005 and 2018, median 2011.

**Table 1:** Radiological characteristics of the RITeS dataset for 668 CTA, assessed by experts.

Radiological feature		*N* (%)
Arterial occlusion^1^	None	303 (45.4%)
	ICA/MCA	341 (50.9%)
	Proximal (ICA‐M1)	256
	Distal (M2+)	85
	Other intracranial artery	24 (3.7%)
	Anterior cerebral	2
	Posterior cerebral	3
	Vertebral	5
	Basilar	5
	Other	9
Collateral scores^2^ (*n* 583)	Good	389 (66.7%)
	Moderate	102 (17.5%)
	Poor	92 (15.8%)
Image quality^2^ (*n* 482)	Good	247 (51.2%)
	Moderate	204 (42.3%)
	Poor	31 (6.4%)
CT slice thickness^2^ (*n* 561)	Thin (≤1 mm)	499 (88.9%)
	Medium (>1 to ≤5 mm)	62 (11.1%)

^1^ Principal abnormality identified, 9 patients had occlusion of both ICA/MCA axis and another intracranial artery.

^2^ Data are not available for all patients, total *N* presented.

### Image processing

Of 668 CTAs uploaded to e‐CTA, 545 (81.6%) were successfully processed. Reasons for not processing 123 CTA (at first attempt, we did not record reasons presented on subsequent attempts): Upload failure (107 CTA), Cancellation of Processing (14 CTA) and Failure of scoring/Failure of segmentation (1 CTA for each). The median time to process a single scan (not including upload) was 125 sec, interquartile range 114–158 sec.

### Primary outcomes

#### Intracranial arterial occlusion detected by e‐CTA compared to experts

From 668 CTA, experts identified arterial occlusion on 365 (54.6%) while e‐CTA found 280 occlusions (51.4% of 545 successfully processed). Of 545 CTAs scored by both experts and e‐CTA, 344 (63.1%) matched exactly: normal (183), laterality‐matched ICA/proximal MCA (149) or distal MCA (12) occlusion. Thirty‐four (6.2%) matched side but differed in proximal‐distal location of ICA‐MCA occlusion. Therefore, 378/545 (69.4%) agreed on the presence/absence of laterality‐matched LVO. Clinically relevant mismatches included disagreement for the presence/absence (121, 22.2%) or laterality (12, 2.2%) of ICA/MCA occlusion. Few mismatches (22, 4.0%) related to vessels outside the scope of software (Table 2). However, after including the 123 CTAs not processed by software, the intention‐to‐analyse proportion of expert‐software agreement for LVO detection drops to 56.6% (378/668).

**Table 2:** Crosstabulation of expert and e‐CTA scoring for identification and location of intracranial arterial occlusion on CTA.

	e‐CTA results						
Expert opinion	Normal	Left ICA/proximal MCA	Left distal MCA	Right ICA/proximal MCA	Right distal MCA	Uncertain^1^	Total
Normal	**183**	13	17	12	10	5	240
Left ICA/proximal MCA	13	**73**	9	6	1	1	103
Left distal MCA	25	12	**7**	0	0	2	46
Right ICA/proximal MCA	16	3	2	**76**	4	4	105
Right distal MCA	10	0	0	9	**5**	5	29
Other artery	18	0	1	0	0	3	22
Total	265	101	36	103	20	20	545

Matched expert and e‐CTA results are in bold.

^1^ Collateral score defect without a corresponding arterial occlusion.

#### Scoring of MCA collaterals by e‐CTA compared to experts

Of 465 CTAs with expert and software collateral scoring, we found maximum expert‐software agreement by combining the ‘poor collaterals’ and ‘no collaterals’ categories of e‐CTA, Table S2. Using this 3‐point reduced score, 325/465 (70%) of collateral scores provided by experts and software matched. Of 140 with mismatched collateral scores: 11 (2%) CTAs had abnormal scores from both software and experts but in opposing cerebral hemispheres; 61 (13%) CTAs were scored by software as having poorer collaterals, 68 (15%) as having better collaterals compared with expert scores for the same hemisphere. As a potential threshold for treatment eligibility, and considering only results in the same hemisphere, 26 (6%) CTAs were scored as having good‐moderate collaterals by experts but downgraded to poor collaterals by software, while 33 (7%) CTAs were conversely upgraded by software to good‐moderate from poor collateral scores provided by experts. The intention‐to‐analyse proportion of expert‐software agreement for collateral scoring was also reduced at 55.7% (325 of 583 provided by experts).

#### Diagnostic accuracy of e‐CTA for detecting arterial abnormality

**Figure 2:**
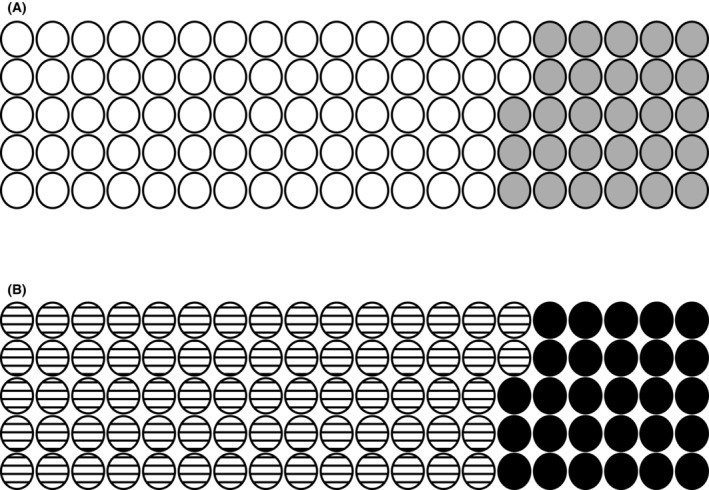
Potential real‐world implications of diagnostic accuracy results for e‐CTA identification of acute angiographic abnormality (arterial occlusion and/or abnormal collateral scoring) compared to masked expert reference standard. (A) Patients with angiographic abnormality. True positive = white. False negative = grey. (B) Patients without angiographic abnormality. True negative = horizontal lines. False positive = black. These analyses include 4% of cases with imaging features outside the intended use of software: non‐ICA/MCA occlusion.

For the detection of any intracranial arterial abnormality (LVO detection and collateral scoring combined), among all patients with stroke symptoms (i.e. not just those with confirmed anterior circulation ischemic stroke), the diagnostic accuracy of e‐CTA compared to experts was as follows: sensitivity 72%, specificity 72% and accuracy 72% (Table 3). Meta‐analysis modelling of individual RITeS studies provided almost identical results with minimal heterogeneity within and between RITeS studies (Figure S1). Figure 2 conveys these results per 100 patients scanned:

- For 100 patients with arterial occlusion assessed using e‐CTA, this will be correctly detected in 72 but missed in 28.
- For 100 patients without arterial occlusion assessed using e‐CTA, this will be incorrectly detected in 28.

**Table 3:** Diagnostic accuracy testing for e‐CTA.

Test	Comparator	Population	*n*	TP	TN	FP	FN	Sensitivity	Specificity	Positive predictive value	Negative predictive value	Accuracy
Identification of any arterial abnormality (LVO or collateral score)	Masked expert at baseline	Full representative cohort	545	209	183	71	82	72 (66–77)	72 (66–77)	75 (70–78)	69 (65–73)	72 (68–76)
		Sensitivity analysis excluding non‐ICA/MCA occlusion and slice thickness >1 mm	466	180	173	54	59	75 (69–81)	76 (70–82)	77 (72–81)	75 (70–79)	76 (72–80)
		Sensitivity analysis, side affected by stroke										
		Not provided to software	259	104	74	43	38	73 (65–80)	63 (54–72)	71 (65–76)	66 (59–73)	69 (63–74)
		Provided to software	286	105	109	28	44	70 (62–78)	80 (72–86)	79 (73–84)	71 (66–76)	75 (69–80)
Identification of LVO only		Full representative cohort	545	195	188	65	97	67 (61–72)	74 (68–80)	75 (71–79)	66 (62–70)	70 (66–74)

Sensitivity, specificity, positive/negative predictive and accuracy results are provided as % (95% CI).

In a sensitivity analysis excluding the 79 patients with non‐ICA‐MCA occlusion or CT slice thickness >1 mm (compliant with the intended use of software): the diagnostic accuracy of e‐CTA compared to experts was non‐significantly improved (*P* = 0.167): sensitivity 75%, specificity 76% and accuracy 76%. In addition, for detecting LVO alone (i.e. regardless of collateral scores) the diagnostic accuracy of e‐CTA was as follows: sensitivity 67%, specificity 74% and accuracy 70% (Table 3).

### Secondary outcomes

#### Factors influencing scan processing success and diagnostic accuracy

Successful CTA processing was associated with younger patient age and more recent year of CTA acquisition, that is less successful for older patients (median +10 years, *P* = 0.005) and for older CTAs (median −2 years, *P* < 0.001). There were trends towards successful processing being associated with patients who presented to hospital earlier or who had a less severe stroke, but these differences were not significant. We did not find that CT slice thickness or reported image quality were significantly associated with processing outcomes, but the thinnest slice scans of good‐moderate quality were more commonly successfully processed (Table 4).

**Table 4:** Univariate comparisons of CTA scans successfully versus unsuccessfully processed by e‐CTA, *n* = 668.

Variable	Successfully processed *n* = 545	Not successfully processed *n* = 123	Absolute difference	*P* value
Age, years	68 (66–81)	78 (66–84)	10 years	0.005
Sex, female	268 (49.2%)	64 (52.0%)	2.8%	0.567
Time from stroke onset, h^1^ (631)	2.1 (2.0–3.3)	2.7 (1.8–4.1)	0.6 h	0.085
NIHSS^1^ (662)	8 (6–16)	12 (6–16)	4	0.092
Scan year	2012 (10–16)	2010 (09–11)	2 years	<0.001
Incidental findings on CT	18 (3.3%)	6 (4.9%)	1.6%	0.397
CT slice thickness^1^ (561)				
≤1 mm	486 (89.2%)	13 (81.2%)	8.0%	0.319
>1–≤5 mm	59 (10.8%)	3 (18.8%)	8.0%	
Image quality^1^ (482)				
Good	186 (51.1%)	61 (51.7%)	0.6%	0.294
Moderate	158 (43.4%)	46 (39.0%)	4.4%	
Poor	20 (5.5%)	11 (9.3%)	3.8%	

Results are median (interquartile range, IQR) or *n* (%) as appropriate. NIHSS, National Institutes of Health Stroke Scale.

^1^ Incomplete data, (available *n*).

On multivariable binary logistic regression with diagnostic accuracy data for e‐CTA detection of any arterial abnormality as the dependent variable (i.e. true positive or true negative versus false positive or false negative), only NIHSS and software knowledge of the side affected by stroke were significantly associated with the extent of expert‐software agreement for LVO detection. Experts and software were more likely to agree when a stroke was more severe (odds ratio = 1.07, 95% CI = 1.03–1.12, *P* = 0.001) and when software was provided with the side of stroke symptoms (odds ratio = 1.82, 95% CI = 1.02–3.24, *P* = 0.041) (Table 5). When the side of stroke symptoms is provided, the diagnostic accuracy improves to 75%, compared with 69% when no side information is given. This change is mostly driven by increased specificity of software (Table 3).

**Table 5:** Binary logistic regression testing factors associated with improved e‐CTA diagnostic accuracy, *n* = 358.

Predictor variables	Raw data	Odds ratio	95% CI	Variance inflation factor	*P* value
Age (years)	68 (66–81)	1.00	0.98–1.02	1.08	0.952
NIHSS	6 (6–16)	1.07	1.03–1.12	1.18	0.001
Time from stroke onset (h)	2.1 (2.0–2.7)	1.02	0.83–1.26	1.05	0.826
e‐CTA had knowledge (Y) of affected side (vs. *N*)	Y = 254 (71%)	1.82	1.02–3.24	1.23	0.041
CT slice thickness (mm)	1.0 (0.62–1.0)	0.83	0.63–1.09	1.06	0.175
Good image quality (vs. moderate‐poor)	Good = 185 (52%)	1.30	0.81–2.09	1.02	0.274

Dependent variable was derived from diagnostic accuracy data for e‐CTA detection of any laterality‐matched arterial abnormality (i.e. true positive or true negative versus false positive or false negative for detection of LVO and/or abnormal collateral score combined). Raw data are median (IQR) or *n* (%), as appropriate. NIHSS, National Institutes of Health Stroke Scale.

### Repeatability testing

Despite initial successful software processing, attempted repeat processing for 2 CTAs was unsuccessful; e‐CTA cited invalid series error for both (both from the older IST‐3 trial). Of 18 CTAs that were successfully reprocessed under identical conditions, there were no differences in results. However, we identified that the presence versus absence of a concurrent non‐enhanced CT did modify e‐CTA results (Table 6).

**Table 6:** Results of e‐CTA repeatability testing with/out concurrent non‐enhanced brain CT, *n* = 20.

Number of cases	With concurrent NECT	Without concurrent NECT
1	Collateral score 3 (normal)^1^	Collateral score 2 (abnormal)
1	ICA/MCA occlusion^1^	No ICA/MCA occlusion
2	Successfully processed	Did not successfully process
6	Minor differences in vessel density (%) without change in collateral score^2^	
	98.8	100
	33.9	30.5
	73.1	69.9
	77.0	76.0
	52.5	57.5
	91.8	92.5
10	No change	

NECT, non‐enhanced CT.

^1^ Matched expert opinion.

^2^ e‐CTA collateral scoring = 3—Excellent (>90%), 2—Good (50–90%), 1—Poor (10–50%), 0—None (0–10%).

## DISCUSSION

We provide independent diagnostic accuracy testing of one commercially available AI software designed to assist clinicians with decision support for acute CT angiography in stroke. We compared e‐CTA software results with separately acquired expert interpretation of the same scans and found that for every 10 patients assessed using e‐CTA, software and expert results for LVO detection and collateral scoring matched (or were clinically equivalent) in seven but disagreed in three. We also found that when LVO detection and collateral scoring were considered together, the diagnostic accuracy of software for detecting acute arterial abnormality in a clinically representative population of patients with presumed ischemic stroke was 72%. This result improved to 76% accuracy if we excluded patients with arterial occlusion in locations not assessed by software or where CT slices were thicker than recommended. In other words, when used as intended, software is likely to correctly detect three quarters of ICA/MCA LVO but may incorrectly detect LVO in one quarter of patients without LVO. A major clinical implication of our results is that one quarter of patients potentially eligible for thrombectomy may not be identified by the software; if the reviewing clinician also overlooks the LVO, these patients could be denied or face delays to highly effective treatment. Additionally, e‐CTA did not successfully process 18% of our scans, which lessens the potential clinical benefit of software.

Three previous studies have assessed the diagnostic accuracy of e‐CTA, two for LVO detection and one for collateral scoring. These studies presented more favourable results for e‐CTA than ours using similar reference standards and including distal occlusions (to M2) as we have done: Seker found 84% sensitivity and 96% specificity for e‐CTA detection of laterality‐matched ICA‐MCA occlusion in 301 patients;^7^ Mallon found 70% sensitivity and 96% specificity for e‐CTA detection of ICA‐MCA occlusion in 83 patients (but did not specify that laterality was matched);^8^ while Grunwald found 99% sensitivity and 94% specificity for e‐CTA detection of favourable collateral scores in 98 patients.^6^ By combining LVO detection and collateral scoring into a single metric, we might expect our results to be at least equivalent to studies looking at these imaging features in isolation. However, these prior studies had more restrictive patient selection than ours by excluding poorer quality or complicated studies (e.g. with spontaneous recanalisation and previous territorial infarct). Finally, only one of these studies was conducted independent of Brainomix.^8^

We found that software was more likely to successfully process scans from younger patients and when CTA acquisition was more recent. Patient age and processing success may relate to the likelihood of older patients having other abnormalities on their scans or being less able to lie still for imaging, although we did not find an association with CTA quality. CTA age and processing success presumably relate to technical differences in older versus newer CTAs and might have been avoided with a prospective study design. Thinner image slices and better image quality may also increase the likelihood of successful processing and have the benefit of being modifiable.

For detecting any arterial abnormality, we found the diagnostic accuracy of e‐CTA improved for the CTAs of patients with more severe stroke, presumably since arterial obstruction in these patients is likely sited proximally and is thus larger and easier to detect. Importantly, the other factor we found to improve the diagnostic accuracy of e‐CTA is user‐modifiable; software knowledge of the side affected by symptoms improved the diagnostic accuracy of software to 75% compared with 69% when this information is not provided. Users should endeavour to manually apply side information for all e‐CTA cases and be aware that results for patients with milder stroke may be less accurate.

In a small test of repeatability, we identified that the presence of a concurrently acquired non‐enhanced CT brain scan caused differences in e‐CTA results compared to software analysis without access to non‐enhanced CT. According to Brainomix, e‐CTA uses concurrent CT to improve localisation of the ICAs as they pass through skull base. This is unlikely to have clinical practice implications (unless centres use e‐CTA to compare baseline and follow‐up scans and only one of these scans has concurrent non‐enhanced CT) but may be important for future research. Finally, in 2 of 20 cases, an attempted repeat test was unsuccessful. Interval modification of unique scan information in our method may be responsible, but we were careful to replace true CTA data with similar dummy data.

### Strengths and limitations of RITeS

According to our pre‐specified standard, RITeS CTAs are representative of the real‐world case mix for whom e‐CTA may be used, including a small proportion of patients with imaging features outside the designated scope of software.^10^ We deliberately included a representative proportion of patients with LVO in non‐ICA‐MCA locations, despite manufacturer guidance that software should only be used to assess patients with anterior circulation stroke since it is not always possible to determine the affected vascular location clinically. Given the order in which events occur routinely (patients with suspected stroke first undergo clinical assessment, followed by imaging, which together are used to differentiate anterior and posterior circulation ischemia) there is nothing to prevent software being used to assess imaging of patients with posterior circulation ischemia. Therefore, it is important to understand the implications of software use in this real‐world context. We provide more clinically meaningful diagnostic accuracy figures by considering all patients who present with suspected acute ischemic stroke rather than only a subset as most previous assessments of AI software for LVO detection have done. We acknowledge that e‐CTA is not designed to be used in isolation of other clinical information or as a standalone diagnostic tool. The guidance for intended use of e‐CTA suggests that competent users should disregard inappropriately acquired software results (e.g. where there is posterior circulation occlusion, an alternative structural diagnosis for stroke symptoms, or in patients without stroke symptoms) but how effectively this is achieved can (and should) only be assessed prospectively.

The proportion of scans not successfully processed in our study is likely to be higher than in scenarios where a local server is used since upload failure to the cloud was most common in RITeS (107/123 failures). A small study that also manually uploaded existing scans to the e‐CTA cloud had fewer failures (2/85, 2.4%) but pre‐emptively excluded scans with movement artefacts.^8^ Our results are important wherever cloud processing is used clinically, but such clinical setups should have a dedicated connection between the CT scanner and cloud for real‐time upload of individual scans that may further improve the rate of successful processing compared to the manual batch upload method we used. Brainomix shared 12 months of e‐CTA processing data with us from a single UK site, demonstrating 98.8% successful processing (1684/1704). Ideally, assessment of AI software for radiology would be conducted prospectively and include all eligible patients who present to receiving hospitals equipped with the software of interest. However, our method is highly efficient and provides clinically relevant results that can inform both routine practice and future prospective research. A difference between software and expert interpretation in RITeS that would likely not occur in a prospective approach is the extent to which the two groups were informed of the side affected by stroke, 50% for software but only 28% for experts. This difference may have caused greater variability in expert results while simultaneously favouring software results. A true head‐to‐head comparison would have provided both experts and software with the same information, but it was not feasible to re‐read all the scans.

In a small number of cases (14/545, 3%), experts and software found abnormality in the opposing side of the brain. For diagnostic accuracy testing, we elected to call these false positives for the hemisphere that the experts considered ‘normal’ rather than false negatives for the hemisphere that the experts considered ‘abnormal’, that is we focussed the interpretation on the software output. Different approaches (e.g. calling them all false negative or splitting the cases equally between the two outcomes) would have modified our diagnostic accuracy results, but given the modest numbers of such events, this would not have significantly changed the results. In this scenario and other cases where experts and software disagreed, it is possible that the software was correct rather than the expert. A consensus opinion from several experts would likely have mitigated this problem, but the trials in RITeS were not resourced for this. Similarly, RITeS was not resourced to allow all expert‐software discrepancies to be revisited.

We used expert interpretation of imaging for comparison with software and while this arguably represents the best option for clinical practice, it is not necessarily realistic nor representative of routine care where specialist opinion may not be immediately available to front‐line clinicians assessing a given patient for thrombectomy referral. In addition, we did not test software as a ‘decision‐support’ tool for front‐line clinicians, that is whether software improves clinical decision‐making. Nevertheless, we feel it is appropriate to compare AI software standalone against gold‐standard clinical practice (i.e. experts) since AI software results are expected to be highly accurate. In addition, the expert standard we employed is highly clinically relevant since the final decision for thrombectomy is made by an expert neurointerventionist. Finally, some centres now acquire multiphase CTA which may improve detection of collaterals, especially where this flow is delayed. However, we did not have access to multiphase imaging in RITeS.

### Conclusions

On independent testing using expert interpretation as the reference standard, e‐CTA software had a diagnostic accuracy of 72–76% for detecting arterial abnormalities including LVO and collateral scoring combined. Eighteen percent of our scans were not processed by software using previously acquired imaging but this result may partly reflect the design of RITeS. Our findings highlight that e‐CTA should only be used according to its clinical approval—namely to assist experienced clinicians interpret CTA for stroke. Such users should be capable of independently reviewing CTA (to disregard inappropriate results) and can improve software detection of true lesions by providing information on the side affected by stroke. Users should also be aware that software results may be less accurate in patients with less severe stroke or who are older.

## Conflict of Interest

GM declares one‐off consultancy fees from Canon Medical for annotating stroke CT images for the development of Canon software. KWM is employed by The University of Glasgow and NHS Greater Glasgow and Clyde who have a research agreement with Brainomix to assess implementation of software as part of an NHSX research grant. All other authors declare no relevant COI.

## Statement of independence

Following development of our research plan and sourcing funding, the RITeS Collaboration signed a software licence agreement with Brainomix for use of e‐CTA and paid for software using academic funds. We agreed to distinguish software testing relating to claims made by Brainomix and for software features with regulatory approval from testing outside the ‘indications for software use’ or for features without regulatory approval. To comply with the agreement, a pre‐submission draft of this paper was shared with Brainomix, and we subsequently made minor changes for technical accuracy. Brainomix staff and affiliates were not involved in creation of the RITeS research plan, setting aims, research conduct including obtaining of data, image processing, statistical analysis, interpretation of data or the writing of the paper.

## Supporting information


**Figure S1.** Meta‐analysis modelling for diagnostic accuracy testing of e‐CTA using individual patient data stratified by contributing study, *n* = 545.
**Table S1.** Demographic and clinical data of patients in RITeS, comparison with other datasets.
**Table S2.** Comparison of angiography collateral scores between e‐CTA and masked experts.

**Data S2.** RITeS eCTA STARD‐2015 Checklist.docx.
