# Supplementary material for: Accuracy of artificial intelligence software for CT angiography in stroke
Source: Ann Clin Transl Neurol. 2023 May 19;10(7):1072–82. doi: 10.1002/acn3.51790 (PMC10351662; doi:10.1002/acn3.51790)
Supplement: Supplementary file 1 — Figure S1. Meta‐analysis modelling for diagnostic accuracy testing of e‐CTA using individual patient data stratified by contributing study, n = 545. Table S1. Demographic and clinical data of patients in RITeS, comparison with other datasets. Table S2. Comparison of angiography collateral scores between e‐CTA and masked experts. [file ACN3-10-1072-s001.docx]

**SUPPLEMENTAL MATERIAL**

**Accuracy of artificial intelligence software for CT angiography in stroke**

Grant Mair (MD)^1^, Philip White (MD)^2^, Philip M Bath (DSc)^3^, Keith Muir (MD)^4^, Chloe Martin^1^, David Dye^1^, Francesca Chappell (PhD)^1^, Rüdiger von Kummer (Prof.Dr.med.)^5^, Malcolm Macleod (PhD)^1^, Nikola Sprigg (DM)^2^, Joanna M Wardlaw (MD)^1,6^, for the RITeS Collaboration.

1. Centre for Clinical Brain Sciences, University of Edinburgh, UK
2. Translational and Clinical Research Institute, Newcastle University and Newcastle upon Tyne Hospitals NHS Trust, UK
3. Stroke Trials Unit, Mental Health & Clinical Neuroscience, University of Nottingham, UK
4. Institute of Neuroscience & Psychology, University of Glasgow, UK
5. Department of Neuroradiology, University Hospital, Technische Universität Dresden, Germany
6. UK Dementia Research Institute Centre at the University of Edinburgh, Edinburgh, UK

**Author for correspondence:**

Dr Grant Mair

Centre for Clinical Brain Sciences

Chancellor’s Building

University of Edinburgh

49 Little France Crescent

Edinburgh

EH16 4SB **Email:** [grant.mair@ed.ac.uk](mailto:grant.mair@ed.ac.uk)

UK **Telephone**: +44 131 495 9563

**Includes:** Supplemental Table 1 and Supplemental Figures 1-2

**Supplemental Figure 1.** Meta-analysis modelling for diagnostic accuracy testing of e-CTA using individual patient data stratified by contributing study, n=545.


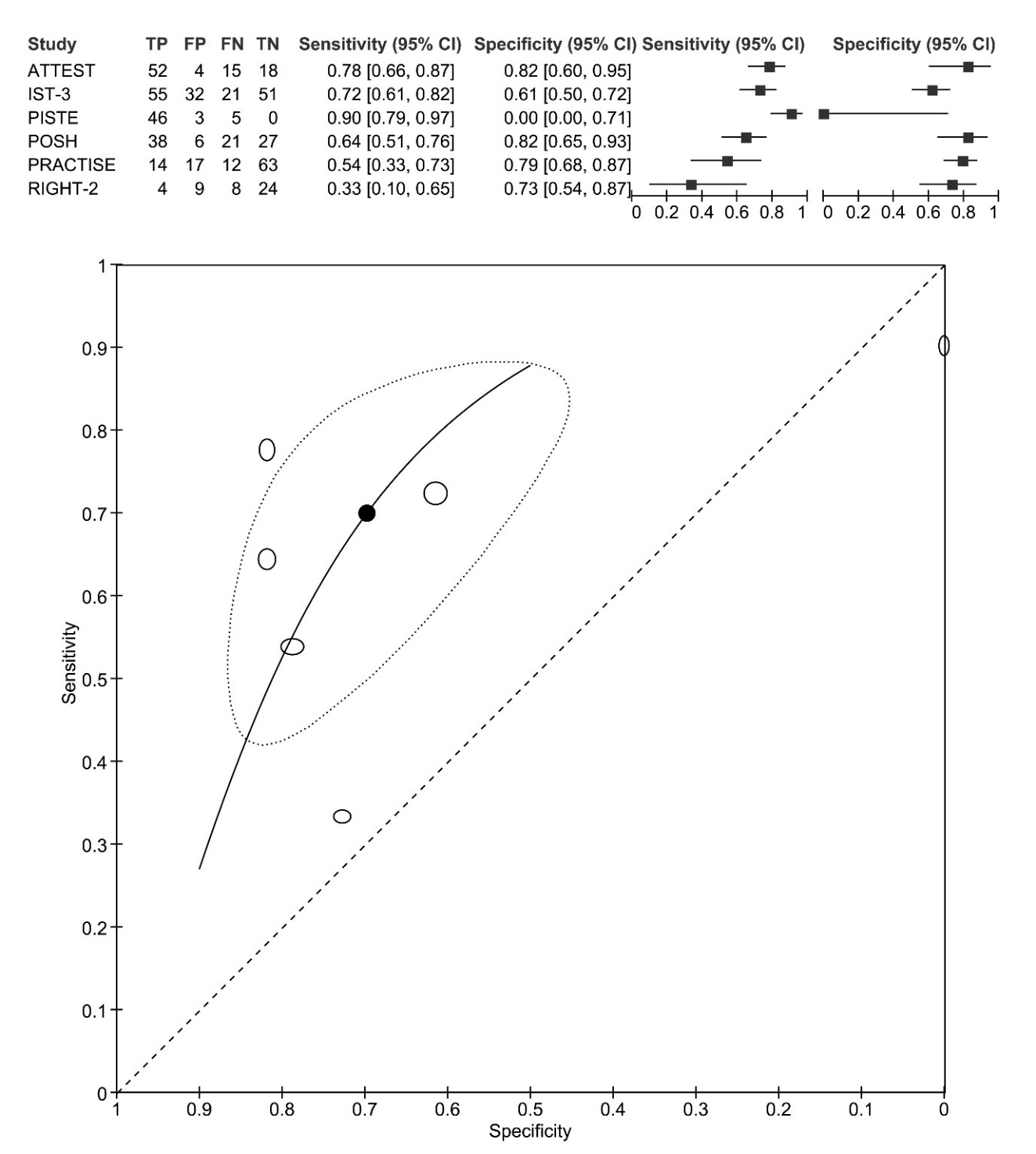


**Note:** Includes patients with non-ICA/MCA occlusion (22/545, 4.0%).

In lower panel bivariate ROC curve, open circles are individual study results proportional to sample size, closed circle is summary result: sensitivity 70% (95%CI 56-81), specificity 70% (57-80). Dotted lines enclose 95% confidence regions.

We used MetaDTA (v2.0: <https://crsu.shinyapps.io/dta_ma/>) to summarise met-analysis data,* and Review Manager (RevMan 5.4, The Cochrane Collaboration) to produce bivariate ROC curves and forest plots.

* Freeman SC, Kerby CR, Patel A, Cooper NJ, Quinn T, et al. Development of an interactive web-based tool to conduct and interrogate meta-analysis of diagnostic test accuracy studies: MetaDTA. *BMC Med Res Methodol.* 2019;19:81

**Supplemental Table 1.** Demographic and clinical data of patients in RITeS, comparison with other datasets.

| **Clinical Feature** | | **RITeS Dataset** | **SSNAP Dataset** | **STTC Dataset** | **HERMES Dataset** |
| --- | --- | --- | --- | --- | --- |
| Total patient number | | 668 | 87,635 | 6,756 | 1,764 |
| Female sex | | 332 (49.7%) | 47.8% | 45% | 47% |
| Age, years | | 71 (66-81) | 77 (66-85) | 71 (13) | 67 (57-76) |
| Cause of stroke symptoms | Ischaemia | 640 (95.8%) | 87.1% | 100% | 100% |
|  | Mimic | 28 (4.2%) | - | - | - |
| NIH Stroke Scale | | 9 (6-16) | 5 (2-11) | 12 (7) | 17 (13-21) |
| Time from stroke onset, hours | | 2.3 (2.0-3.5) | 4 (2-11) | 4 (1.2) | 3 (2-4) |

**Note:** Data are percentage, median (inter-quartile range), or mean (standard deviation) as appropriate.

RITeS clinical variables were within the interquartile ranges, or ±1 SD of the mean, or <5 percentage points for all 3 comparative datasets: except time from stroke onset in STTC.

SSNAP - UK Sentinel Stroke National Audit Programme.

STTC – Stroke Thrombolysis Trialists’ Collaboration.

HERMES – Highly Effective Reperfusion Evaluated in Multiple Endovascular Stroke Trials collaboration.

**Supplemental Table 2.** Comparison of angiography collateral scores between e-CTA and masked experts.

| **e-CTA Modified Tan** | **Miteff Comparison 1** | **Miteff Comparison 2** | **Miteff Comparison 3** |
| --- | --- | --- | --- |
| 3 – Excellent (>90%) | Good | Good | Good |
| 2 – Good (50-90%) |  | Moderate | Moderate |
| 1 – Poor (10-50%) | Moderate |  | Poor |
| 0 – None (0-10%) | Poor | Poor |  |
| **Expert-software collateral score match** | **318/465 (68%)** | **319/465 (69%)** | **325/465 (70%)** |

**Note:** Results based on 465 result pairs.
